# Differentiation between Oppositely Oriented Microtubules Controls Polarized Neuronal Transport

**DOI:** 10.1016/j.neuron.2017.11.018

**Published:** 2017-12-20

**Authors:** Roderick P. Tas, Anaël Chazeau, Bas M.C. Cloin, Maaike L.A. Lambers, Casper C. Hoogenraad, Lukas C. Kapitein

**Affiliations:** 1 Division of Cell Biology, Department of Biology, Faculty of Science, Utrecht University, Padualaan 8, 3584 Utrecht, the Netherlands

**Keywords:** neurons, neuronal polarity, polarized transport, microtubules, motor proteins, kinesin, dendrites, axon, microtubule orientation, microtubule polarity

## Abstract

Microtubules are essential for polarized transport in neurons, but how their organization guides motor proteins to axons or dendrites is unclear. Because different motors recognize distinct microtubule properties, we used optical nanoscopy to examine the relationship between microtubule orientations, stability, and modifications. Nanometric tracking of motors to super-resolve microtubules and determine their polarity revealed that in dendrites, stable and acetylated microtubules are mostly oriented minus-end out, while dynamic and tyrosinated microtubules are oriented oppositely. In addition, microtubules with similar orientations and modifications form bundles that bias transport. Importantly, because the plus-end-directed Kinesin-1 selectively interacts with acetylated microtubules, this organization guides this motor out of dendrites and into axons. In contrast, Kinesin-3 prefers tyrosinated microtubules and can enter both axons and dendrites. This separation of distinct microtubule subsets into oppositely oriented bundles constitutes a key architectural principle of the neuronal microtubule cytoskeleton that enables polarized sorting by different motor proteins.

## Highlights

• Motor-based nanoscopy enables direct observation of microtubule (MT) polarity
• In neurons, MTs organize into polarized bundles that locally bias transport
• In dendrites, bundles of opposite orientation differ in stability and composition
• Dendritic MTs bias Kinesin-1 transport toward the soma, ensuring axon selectivity

## Teaser

Tas et al. use optical nanoscopy to show that dendritic microtubules of opposite orientation differ in stability and composition and recruit different motor proteins. This explains why some motor proteins can move into dendrites while others can only enter axons.

## Introduction

The polarized organization of neurons depends on the selective targeting of cargoes to either axons or dendrites, driven by motor proteins that move selectively toward either the plus or the minus end of microtubules (Bentley and Banker, 2016, Britt et al., 2016, Hirokawa et al., 2010, Kapitein and Hoogenraad, 2011, Stiess and Bradke, 2011, Vale, 2003). For some axonal cargoes, selective targeting is established by non-selective transport to axons and dendrites, followed by selective endocytosis in dendrites, while other cargoes are directly targeted to axons and do not enter dendrites (Bentley and Banker, 2016, Sampo et al., 2003, Wisco et al., 2003). In lower organisms, such as *Drosophila* or *C. elegans*, microtubule orientations in dendrites and axons are both uniform but of opposite orientation (Kapitein and Hoogenraad, 2015, Maniar et al., 2011, Rolls, 2011). Here, plus-end-outward-oriented microtubules in axons enable kinesin-driven anterograde transport, whereas transport into dendrites depends on minus-end-directed motors because the microtubule orientations are reversed (Harterink et al., 2016, Rolls, 2011). In contrast, in dendrites of mammalian neurons, microtubules are equally mixed between both orientations (Baas et al., 1988, Kleele et al., 2014, Yau et al., 2016). Remarkably, several plus-end-directed motor proteins can nevertheless selectively enter axons, while others target both axons and dendrites (Huang and Banker, 2012, Jacobson et al., 2006, Kapitein et al., 2010a, Lipka et al., 2016, Nakata and Hirokawa, 2003). While it is widely assumed that these fundamental differences in selectivity are encoded by the neuronal microtubule network (Janke, 2014, Verhey and Gaertig, 2007), the design principles that ensure axon-selective transport have remained unresolved (Bentley and Banker, 2016, Britt et al., 2016, Kapitein and Hoogenraad, 2011, Stiess and Bradke, 2011).

It has been reported that certain members of the kinesin superfamily can preferentially interact with microtubule subsets that carry specific chemical modifications or associated proteins (Atherton et al., 2013, Cai et al., 2009, Konishi and Setou, 2009, Sirajuddin et al., 2014). For example, the axon-selective Kinesin-1 has been shown to prefer stable microtubules marked by acetylation and detyrosination (Cai et al., 2009, Konishi and Setou, 2009), while the non-selective Kinesin-3 has been suggested to preferentially bind to tyrosinated microtubules (Guardia et al., 2016, Lipka et al., 2016). Nevertheless, these properties alone cannot explain the axon selectivity of Kinesin-1, given that acetylated and detyrosinated microtubules are also abundantly present in dendrites (Hammond et al., 2010). The mild enrichment of these microtubules in proximal axons versus proximal dendrites (Hammond et al., 2010) is insufficient to explain why Kinesin-1 exclusively enters axons and fails to enter dendrites (Kapitein et al., 2010a). Thus, despite the accumulating evidence for and understanding of the selective binding of motors to specific subsets of microtubules, the link between selective microtubule binding and selective axonal entry has remained unclear.

The axon selectivity of the plus-end-directed Kinesin-1 could be explained if the microtubules preferred by this motor would be largely oriented minus-end out in dendrites. Likewise, if the microtubule subset preferred by non-selective motors would be oriented plus-end out, this would explain why these motors can drive anterograde transport in dendrites. To test this, we here use novel optical nanoscopy techniques to dissect the relationship between microtubule orientations, stability, and modifications in neurons. Using nanometric tracking of motor proteins running over an extracted cytoskeleton to super-resolve microtubules and determine their polarity, we find that dendritic microtubules are organized in polarized bundles that locally bias transport. In dendrites, these polarized bundles with opposite orientations differ in overall stability and composition, with minus-end-out microtubules being more stable and more acetylated. We also show that Kinesin-1 selectively binds to these minus-end-out-oriented microtubules, which explains why this plus-end-directed motor cannot drive cargo transport into dendrites. In addition, we find that Kinesin-3 preferentially binds to microtubules that are mostly oriented plus-end out, which explains why this motor can enter dendrites. Thus, the separation of distinct microtubule subsets into oppositely oriented bundles constitutes a key architectural principle of the neuronal microtubule cytoskeleton that enables selective sorting by different motor proteins.

## Results

### motor-PAINT: Super-Resolution Imaging of Microtubules and Their Orientation

**Figure 1:**
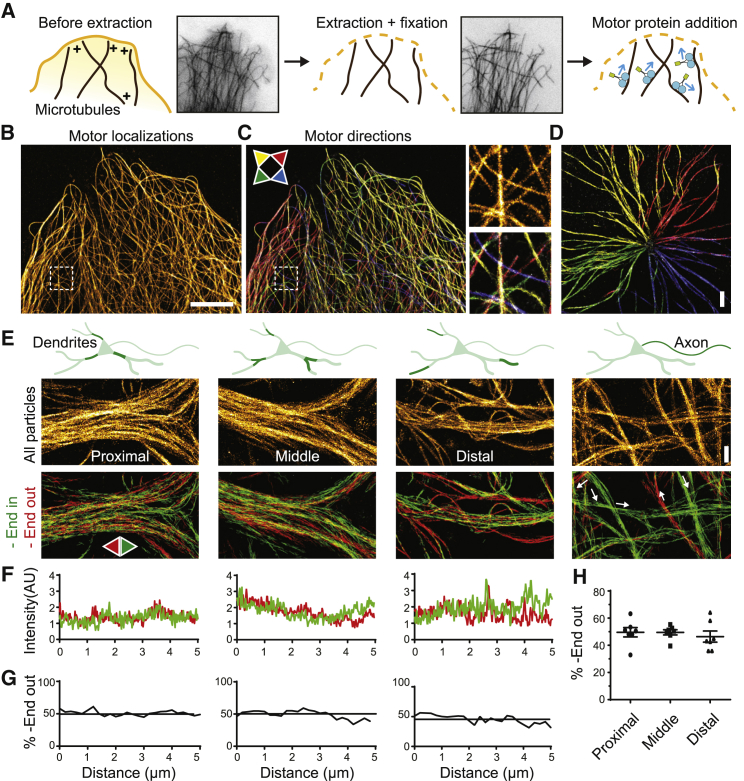
motor-PAINT: Super-Resolution Imaging of Microtubules and Their Orientation (A) Assay: after extraction and fixation, purified and fluorescently labeled motors are added and map out the microtubule array by unidirectional runs. See also Figures S1 and S2. (B) Super-resolved image of an extracted U2OS cell obtained by subpixel localization of thousands of motor binding events. (C) Left: super-resolution reconstruction of the same cell with all microtubule segments colored according to their absolute orientation. Legend arrows point in the direction of the plus end. Right: zooms showing free microtubule plus and minus end. See also Figure S2. (D) Orientation mapping of a centrosomal microtubule array obtained after nocodazole washout in a COS7 cell. (E) Motor-based super-resolution reconstruction of microtubules in dendrites and axons of cultured rat hippocampal neurons (DIV16–DIV17). Top images are based on all binding events (>42,405 events per image). Bottom images are color coded for absolute orientation. Track interpolation was used for all run-based images. (F) Quantification of inward- and outward-moving kinesins in 5-μm-long proximal, middle, and distal dendritic segments, reflecting the number minus-end-outward- and -inward-oriented microtubules, respectively. (G) Percentage of minus-end-out-oriented microtubules in proximal, middle, and distal dendritic segments, based on the graphs shown in (F). (H) Average percentage of minus-end-out-oriented microtubules in proximal, middle, and distal dendritic segments (mean ± SEM, n = 7 segments from 7 neurons for every category). Scale bars, 5 μm (B), 1 μm (D and E).

To test whether axon-selective kinesins only interact with the subset of microtubules that is oriented minus-end out in dendrites, we set out to explore the relation between microtubule orientations, stability, and modifications in neurons. Super-resolution microscopy (Hell, 2007, Huang et al., 2009, Molle et al., 2016, Patterson et al., 2010) enables resolving individual microtubules in dense networks (Mikhaylova et al., 2015) and can also detect specific microtubule modifications, but robust detection of microtubule orientations has not yet been demonstrated. We reasoned that using nanometric tracking of plus-end-directed motor proteins would super-resolve microtubules and also reveal their polarity. Because this approach requires detecting thousands of single-molecule events over the course of several minutes and any concurrent microtubule rearrangement would blur the final image, we tested whether motor proteins could still move over microtubules after chemical fixation of the cytoskeleton (Brawley and Rock, 2009, Sivaramakrishnan and Spudich, 2009) (Figure 1A). COS7 or U2OS cells were permeabilized using detergent, extracted, and fixed using paraformaldehyde, and real-time imaging was used to carefully optimize buffer conditions for extraction and fixation (Figure S1). This resulted in a procedure to successfully preserve microtubule organization without any noticeable rearrangements or depolymerization (Figure 1A; Figures S1 and S2; Movie S1). Subsequent addition of purified and fluorescently labeled kinesin molecules (DmKHC-GFP) resulted in numerous transient events of motors binding to the microtubule and running over it for hundreds of nanometers with a speed of 7.5 ± 3.0 × 10^2^ nm/s (average ± SD) (Figure 1A; Figure S2; Movie S2). Thus, motor proteins can still move over a chemically fixed cytoskeleton to report microtubule orientations.

Next, we analyzed these binding events using single-molecule localization and tracking algorithms to construct a diffraction-unlimited image of the microtubule array in which the absolute orientation of each microtubule is known along the entire microtubule lattice (Figures 1B–1D; Figure S2). Line scans across individual microtubules revealed a full width at half maximum (FWHM) of 52 ± 5 nm (mean ± SD for n = 30 profiles; Figure S2), suggesting a lateral resolution of the same magnitude, because microtubule FWHM is a good predictor of lateral resolution in case of sufficient labeling density (Mikhaylova et al., 2015). To validate the motors’ trajectories as reliable readout of microtubule polarity, we treated cells with the microtubule-destabilizing agent nocodazole. Subsequent washout induced rapid regrowth of microtubules nucleated by the centrosome, resulting in a well-defined radial array of microtubules with all plus ends oriented outward. Indeed, the motor-based super-resolution image obtained for these cells unambiguously confirmed this organization, with 100% of centrosome-associated microtubules attached with their minus end (Figure 1D; 141 microtubules in 3 cells). Thus, motors moving over an extracted cytoskeleton reliably report the polarity of microtubules and can be used to reconstruct a super-resolved image. Because our method is conceptually related to transient binding approaches that can be classified as PAINT variants (point accumulation for imaging in nanoscale topography) (Giannone et al., 2010, Jungmann et al., 2014, Kiuchi et al., 2015, Molle et al., 2016, Sharonov and Hochstrasser, 2006), we termed it motor-PAINT.

### Dendritic Microtubule Arrays Form Bundles of Preferred Polarity

**Figure 2:**
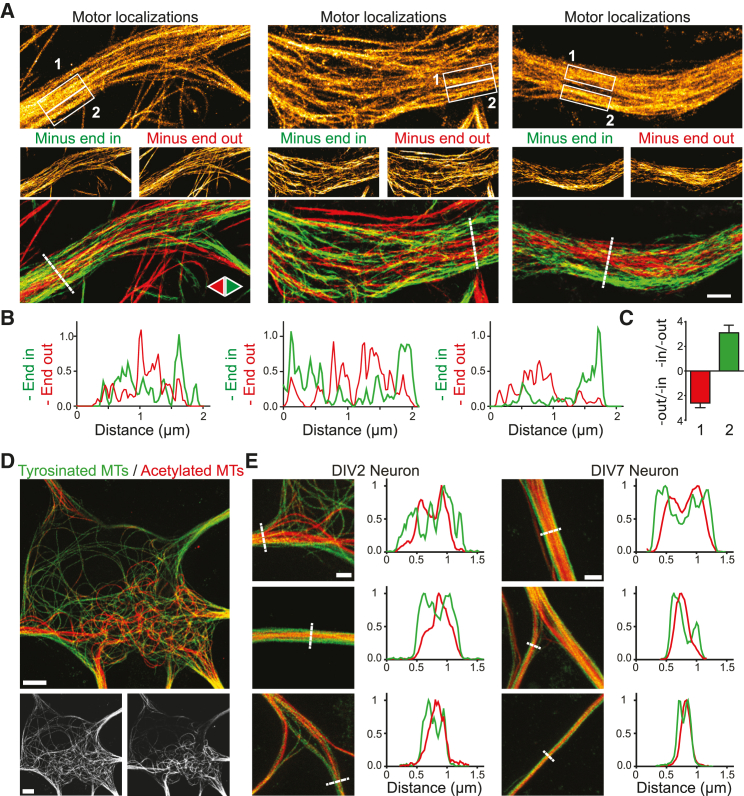
Dendritic Microtubule Arrays Spatially Segregate by Orientation and Modification (A) Three examples of dendrites demonstrating bundles of preferred polarity (left-right). Motor-based super-resolution reconstructions based on all binding events (top), inward runs (middle right), outward runs (middle left), or runs of both directions (bottom). Track interpolation was used for the run-based images. (B) Intensity profiles for inward- and outward-pointing microtubules along the lines indicated in (A). See also Figure S3. (C) Ratio between outward and inward runs or inverse for regions marked with 1 or 2 in (A). Mean ± SEM. (D) STED image from the soma of a DIV2 neuron immunostained for tyrosinated and acetylated MTs (top) and the individual tyrosinated (bottom left) and acetylated channel (bottom right). (E) Zooms from DIV2 and DIV7 neurites highlighting spatial segregation between tyrosinated and acetylated MTs. Corresponding intensity profiles along the indicated line is shown next to the image on the right. See also Figure S4. Scale bars, 1 μm (A and E), 5 μm (D).

We next used our methodology to explore the microtubule organization in the dendrites and axons of rat hippocampal neurons. Consistent with earlier reports (Baas et al., 1988, Stepanova et al., 2003, Yau et al., 2016), microtubules in axons were uniformly oriented, whereas microtubules in dendrites were oriented both ways (Figure 1E; Movie S3). Comparing the number of outward and inward runs in 5 μm stretches in proximal, middle, and distal regions of dendrites revealed that 50% of the microtubules were oriented minus-end out throughout the dendrite (Figures 1E–1H). Interestingly, the separate images created for minus-end-outward- and minus-end-inward-oriented microtubules were not identical. Often, spatially separated bundles of microtubules in dendrites would be enriched for one orientation, indicating local orientational order (Figures 2A and 2B). Spatial correlation analysis and intensity quantification (Figure S3; see STAR Methods) revealed that at lateral length scales below 600 nm, dendritic microtubules are enriched 2- to 4-fold for a specific orientation, suggesting a 66%/33% to 80%/20% ratio between microtubules of opposing orientations (Figure 2C). Thus, the dendritic microtubule array is comprised of bundles of preferred polarity, while, overall, both orientations are equally abundant.

### Minus-End-Out-Oriented Microtubules Are More Stable and More Acetylated

To explore the relation between microtubule modifications and orientations, we first performed nanoscopy of markers for different microtubule subsets. Immunolabeling of tyrosinated tubulin reveals freshly polymerized microtubules in which most α-tubulins have not yet lost their C-terminal tyrosine or their penultimate glutamate, whereas staining for acetylated tubulin or Δ2-tubulin (detecting loss of the C-terminal tyrosine and glutamate) labels stable microtubules (Janke, 2014). STED (stimulated emission depletion) microscopy (Figures 2D and 2E) and localization microscopy (Figure S4) revealed that acetylated microtubules and tyrosinated microtubules form spatially separated subsets both in the cell soma and within dendrites. In addition, tyrosinated microtubules did not overlap with Δ2-tubulin, while the EB1-positive stretches that label growing microtubules were enriched in regions with strong tyrosinated tubulin signals (Figure S4). These results demonstrate that dendritic microtubules spatially segregate into bundles with either dynamic or stable microtubules.

**Figure 3:**
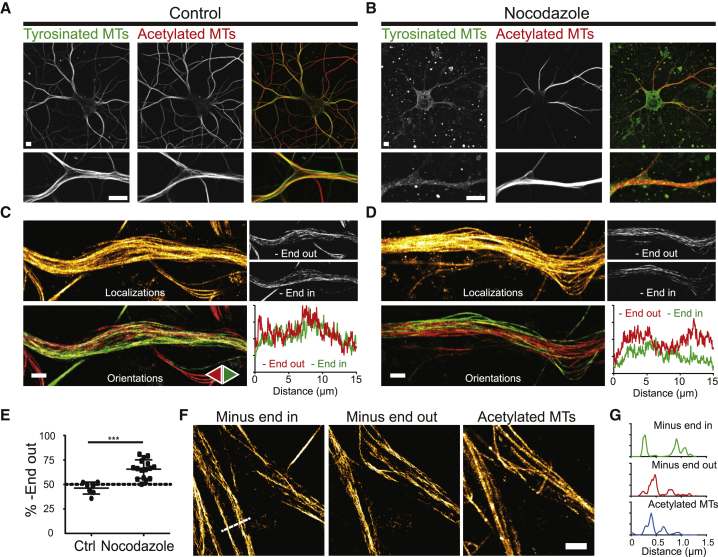
Minus-End-Out-Oriented Microtubules Are More Stable and More Acetylated (A and B) Overview and zoom of DIV9 neurons immunostained for tyrosinated and acetylated microtubules in control conditions (A) or following 2.5 hr incubation with 4 μM nocodazole (B). (C and D) motor-PAINT performed on a dendritic segment in control conditions (C) or after nocodazole treatment (D). (E) Percentage of minus-end-out-oriented microtubules in dendritic segments in control and nocodazole-treated neurons. Mean ± SD, control: n = 7, nocodazole: n = 16 acquired in 3 independent experiments. t test: ^∗∗∗^p < 0.001. (F) Correlative reconstructed images of minus-end in (left), minus-end out (middle), and acetylated microtubules (right) of a dendritic segment. See also Figure S5. (G) Intensity profiles measured for both microtubule orientations and the acetylated tubulin channel along the line indicated in (F). Scale bars, 5 μm (A and B), 1 μm (C, D, and F).

So far, motor-PAINT revealed bundles enriched for plus- or minus-end-out microtubules, while immunolabeling revealed bundles enriched for stable or dynamic microtubules. To resolve the connection between these different bundles and directly explore whether stable microtubules have a preferred orientation, we treated neurons with 4 μM nocodazole for 2.5 hr to selectively preserve stable microtubules and subsequently performed motor-PAINT microscopy. Immunolabeling confirmed the loss of tyrosinated microtubules, while the acetylated microtubule network was still intact (Figures 3A and 3B). motor-PAINT microscopy on the remaining network of stable microtubules revealed that minus-end-out microtubules were approximately twice as abundant as plus-end-out microtubules (66% versus 34% for plus- and minus-end out, respectively; Figures 3C–3E). In addition, to explore the link between microtubule stability and orientation without microtubule destabilization, motor-PAINT microscopy on unperturbed networks was followed by immunolabeling of acetylated tubulin, which revealed that acetylated microtubules predominantly overlapped with minus-end-out-oriented microtubules (Figures 3F and 3G; Figure S5). Together, these results demonstrate that the spatially segregated stable and dynamic microtubules networks are enriched in minus-end-out- and plus-end-out-oriented microtubules, respectively.

### Kinesin-1 and Kinesin-3 Prefer Different Microtubule Subsets in Neurons

**Figure 4:**
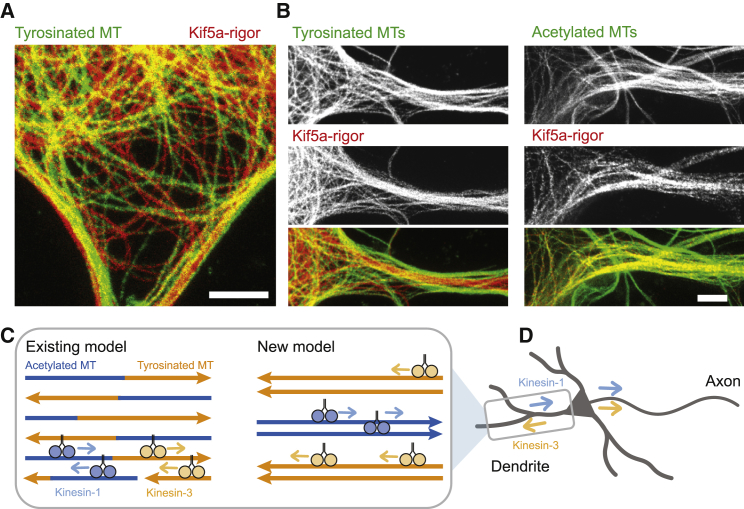
Kinesin-1 Prefers Stable, Acetylated Microtubules in Neuronal Axon and Dendrites (A and B) STED image of DIV4 polarized neuronal soma (A) or neurites (B) after 1 day expression of GFP-kif5a rigor stained for GFP and acetylated-tubulin or GFP and tyrosinated-tubulin. See also Figure S6. (C) Cartoon illustrating the existing and new model for dendritic microtubule organization. Arrowheads on microtubules depict plus ends. Kinesin-1 and Kinesin-3 preferentially move over stable/acetylated and dynamic/tyrosinated microtubules, respectively. (D) The new model can explain the selective entry of Kinesin-1 into axons. Arrows depict bias in transport directionality (see also Figure S7). Scale bars, 2 μm (A and B).

Kinesin-1 has been shown to prefer stable microtubules marked by acetylation and detyrosination (Cai et al., 2009, Konishi and Setou, 2009), while the non-selective Kinesin-3 has been suggested to preferentially bind to tyrosinated microtubules (Guardia et al., 2016, Lipka et al., 2016). Together with our findings that stable and dynamic microtubules are predominantly oriented minus-end outward and plus-end outward, respectively, this would explain why Kinesin-1 cannot enter dendrites, while Kinesin-3 enters dendrites and accumulates at their tips. Nevertheless, the exact binding bias of Kinesin-1 and Kinesin-3 in neurons has not yet been determined. To measure this, we overexpressed a rigor mutant of Kinesin-1 (Kif5a-rigor), which can bind to microtubules but neither walk nor detach (Farías et al., 2015). Kinesin-1 showed a very dramatic colocalization with acetylated tubulin, whereas staining for tyrosinated tubulin highlighted a microtubule network that was completely devoid of Kinesin-1 (Figures 4A and 4B; Figure S6). At moderate overexpression levels, the motor was largely bound to acetylated/non-tyrosinated microtubules in the cell body and near the axon entry, whereas at very high levels, it was also present in dendrites but still selective for acetylated/non-tyrosinated microtubules (Figures 4A and 4B). Thus, Kinesin-1 selectively binds to those dendritic microtubules whose plus ends are mostly oriented toward the cell body, which prevents entry into dendrites and ensures axon selectivity.

For Kinesin-3, we found that a rigor construct (Kif1a-Rigor-GFP) (Guardia et al., 2016) was 1.5- to 2-fold enriched on tyrosinated microtubules versus acetylated microtubules in COS7 cells (Figures S6D–S6F). Because this motor is not completely excluded from acetylated microtubules, assessing preferential binding in neurons was challenging given the high density of microtubules. However, in somatic regions where tyrosinated and acetylated microtubules were clearly separated, we could analyze the binding preference and again found a 1.5- to 2-fold enrichment on tyrosinated microtubules (Figures S6G and S6H). Given that tyrosinated microtubules are preferentially oriented plus-end out, this enrichment is sufficient to bias transport to the distal end (Figure S6I). As shown in Figure S7, even a small difference in outward versus inward runs (e.g., P_outward_ = 0.55–0.6) will already direct most motors to the distal end of the dendrite (Figure S7).

## Discussion

Existing models of the dendritic microtubule array suggest that microtubules of different orientations are randomly distributed and have a similar composition, often consisting of a stable, chemically modified segment followed by a dynamic, tyrosinated end (Figure 4C) (Baas et al., 2016, Conde and Cáceres, 2009, Nirschl et al., 2017). We here used novel optical nanoscopy techniques to dissect the relation between microtubule orientations, stability, and modifications in neurons (Balabanian et al., 2017). This revealed unanticipated local orientational order in dendrites where microtubules are organized in multiple polarized bundles, whose properties depend on their absolute orientation. Minus-end-out-oriented microtubules are more stable and more modified, while the plus-end-out microtubules are more dynamic.

The finding that the microtubules preferred by Kinesin-1 are predominantly oriented minus-end out in dendrites solves how this motor can be axons selective, while the microtubules it prefers are also found in dendrites (Figures 4C and 4D). In addition, the finding that dynamic microtubules are largely oriented plus-end out explains why motors that preferentially interact with these microtubules, such as Kinesin-3, not only enter dendrites (Jacobson et al., 2006, Kapitein et al., 2010a, Nakata and Hirokawa, 2003), but also accumulate at dendrite tips (Huang and Banker, 2012, Jacobson et al., 2006, Lipka et al., 2016) (Figures 4C and 4D). Our results thus support the tubulin-code hypothesis by showing that microtubules with different modifications and associated proteins have different functional roles (Janke, 2014, Verhey and Gaertig, 2007).

In addition, for motors without a preference for specific microtubule subsets, the bundles of uniform polarity that we identified promote persistent motility in a certain direction, because once moving within a bundle, motility will be biased despite potential switching between different microtubules within a bundle. Mathematical modeling revealed that even a small asymmetry will create a directional bias for motors (Kapitein et al., 2010a) (Figure S7). For example, when plus-end-out microtubules are 2-fold enriched, 50% of kinesin-driven cargoes would accumulate in the last 20% or 9% of the bundle for a bundle length of 20 or 50 μm, respectively (Figure S7).

Given that dendrite-entering kinesins can also enter axons, additional mechanisms are required to prevent axonal entry of dendritic cargoes, which are most likely mediated by the minus-end-directed microtubule motor dynein and by myosin motors that can oppose microtubule-based transport (Arnold and Gallo, 2014, Kapitein et al., 2010a, Kapitein et al., 2013, Kuijpers et al., 2016, Watanabe et al., 2012), possibly augmented by specific filtering properties of the axon initial segment (Leterrier and Dargent, 2014, Rasband, 2010, Song et al., 2009). Furthermore, how motors selectively recognize specific microtubule subsets remains poorly understood. Current models implicate direct effects of different modifications or important roles for different microtubule-associated proteins (MAPs) in loading motors to specific microtubules (Atherton et al., 2013, Bentley and Banker, 2016, Janke, 2014, Lipka et al., 2016). In addition, the mechanisms by which the sophisticated organization of dendritic microtubules is established are unknown, but they likely depend on a multitude of MAPs and microtubule-organizing motors that collectively phase separate and polarity sort different microtubule populations (Kapitein and Hoogenraad, 2015). Regardless of the mechanisms, our finding that microtubules with opposite orientations differ in stability and chemical composition constitutes a key architectural principle of the neuronal microtubule cytoskeleton that enables polarized sorting by different motor proteins.

## STAR★Methods

### Key Resources Table

**Table:** 

REAGENT or RESOURCE	SOURCE	IDENTIFIER
**Antibodies**		

Mouse anti-Acetylated tubulin	Sigma	Cat#T7451, RRID: AB_609894
Rat anti-Tyrosinated tubulin	Abcam	Cat#Ab6160, RRID: AB_305328
Rabbit anti-Δ2 tubulin	Millipore	Cat# AB3203, RRID: AB_177351
Mouse Anti-EB1	BD Bioscience	Cat#610535, RRID: AB_397892
Rabbit anti-GFP	MBL	Cat#598S, RRID: AB_591816
Alexa 647 Goat Anti-Rat IgG (H+L)	Molecular Probes, Life Technologies	Cat#A212417
Alexa 488 Goat Anti-Rat IgG (H+L)	Molecular Probes, Life Technologies	Cat#A11006, RRID: AB_141373
Alexa 647 Goat Anti-Mouse IgG (H+L)	Molecular Probes, Life Technologies	Cat#A21236, RRID: AB_141725
Alexa 594 Goat Anti-Mouse IgG (H+L)	Molecular Probes, Life Technologies	Cat#A11032, RRID: AB_141672
Alexa 568 Goat Anti-Mouse IgG (H+L)	Molecular Probes, Life Technologies	Cat#A11031, RRID: AB_144696
Alexa 568 Goat Anti-Rabbit IgG (H+L)	Molecular Probes, Life Technologies	Cat#A11036, RRID: AB_143011
Alexa 594 Goat Anti-Rabbit IgG (H+L)	Molecular Probes, Life Technologies	Cat#A11032, RRID: AB_141672

**Chemicals, Peptides, and Recombinant Proteins**		

ATP	Sigma	Cat# A2383-5G
Neurobasal	GIBCO	Cat#21103-049
B27	GIBCO	Cat# 17504044
cOmplete protease inhibitor cocktail	Roche	Cat#11836145001
cOmplete His-Tag Purification Resin	Roche	Cat#11898400
Superdex 75 10/300 gel filtration Colum	GE Healthcare	Cat#17-5174-01
Paraformaldehyde (16%)	EMS	Cat#15710
Paclitaxel	Sigma	Cat#T7402-25MG
Nocodazole	Sigma	Cat#M1404
Catalase	Sigma	Cat# C40
Glucose oxidase	Sigma	Cat# G1233
β-Mercaptoethylamine (MEA)	Sigma	Cat# 30070
ProLong Diamond antifade mountant	Thermo Scientific	Cat#P36970
Purified DmKHC(1-421)-GFP-6xHis	This paper	N/A

**Critical Commercial Assays**		

Lipofectamine2000	Invitrogen	Cat#1639722
FuGENE 6	Roche	Cat#11836145001

**Experimental Models: Cell Lines**		

Monkey: Cos7	Laboratory of Anna Akhmanova	N/A
Human: U2OS	ATCC	ATCC
Rat: embryonic day 18 hippocampal primary neuron culture	N/A	N/A

**Experimental Models: Organisms/Strains**		

*Escherichia coli*: BL21DE3	N/A	N/A

**Recombinant DNA**		

Plasmid: DmKHC(1-421)-GFP-6x His	This paper	N/A
Plasmid: GFP-Kif5a-rigor	Farías et al., 2015	N/A
Plasmid: Cherry-Tubulin	This paper	N/A

**Software and Algorithms**		

ImageJ	NIH	https://imagej.nih.gov/ij/; RRID: SCR_003070
Fiji	Fiji	http://fiji.sc; RRID: SCR_002285
GraphPad Prism	GraphPad Software	https://www.graphpad.com/scientific-software/prism/; RRID: SCR_002798
Python(x,y)/Spyder3	The Spyder Project Contributors	http://python-xy.github.io/; RRID: SCR_006903
Track allocation algorithm	This paper	N/A

### Contact for Reagent and Resource Sharing

Further requests for information and for resources and reagents should be directed to and will be fulfilled by the Lead Contact, Lukas Kapitein (l.kapitein@uu.nl).

### Experimental Model and Subject Details

#### Cell Lines and Tissue Culture

COS7 cells were cultured in DMEM/Ham’s F10 (1:1) medium containing 10% FCS and penicillin/streptomycin.

Primary hippocampal cultures were prepared from embryonic day 18 (E18) rat brains (Kapitein et al., 2010b). Cells were plated on coverslips coated with poly-L-lysine (37.5 μg ml^−1^) and laminin (1.25 μg ml^−1^). Hippocampal cultures were grown in Neurobasal medium (NB) supplemented with 2% B27 (Invitrogen), 0.5 mM glutamine, 15.6 μM glutamate, and 1% penicillin plus streptomycin.

The sex of cells was not determined.

### Method Details

#### DNA Constructs and Protein Purification

To generate DmKHC(1-421)-GFP-6xHis, amino acids 1-421 of the *Drosophila* kinesin heavy chain were inserted in a pET28a-GFP-6xHis expression vector in the NcoI and EcoRI site. GFP was previously inserted between the EcoRI and XhoI sites. The construct was verified by sequencing and transformed in the BL21DE3 bacterial strain. The GFP-Rigor-KIF5A cloned into a peGFP vector was a gift from Ginny Farías (Farías et al., 2015). pβactin-Kif1a-FRB was described previously (Lipka et al., 2016). Kif1a-Rigor-GFP-FRB was cloned by PCR of the N terminus (AA1-253) and the fused C terminus (AA253-383-GFP) of Kif1a, substituting glutamic acid to lysine at amino acid 253. Both fractions were fused through Gibson assembly (addgene) and ligated into pβactin restricted with AscI/SpeI.

To express the DmKHC(1-421)-GFP-6xHis, a 2L culture was grown until OD0.6. Expression was induced with 1mM of IPTG and cells were grown for 0.5 hours at 37°C and 3.5 hours at 20° C. Cells were then pelleted by centrifugation and resuspended on ice in resuspension buffer (20mM Pipes, 150mM NaCL, 4 mM MgSO4, pH7.0) supplemented with lysozyme and protease inhibitor cocktail (Roche). Subsequently, cells were lysed through 5 rounds of 30 s sonication. The soluble fraction containing the expressed protein was separated through 40 minutes centrifugation at 20000 g and incubated with NiNTA beads (Roche) for 1 hour at 4°C.

Beads were washed 3 times in resuspension buffer supplemented with 50 μM ATP and in the last wash 60mM imidazole was added. Recombinant protein was eluted for 15 minutes in Elution buffer (80mM Pipes, 4mM MgSO_4_, 300mM imidazole, 50μM ATP, pH7.0). The supernatant was concentrated to 0.5 mL and recombinant protein was further purified and buffer exchanged through gel filtration on a superdex75 column (GE Healthcare, Superdex 75 10/300) equilibrated with PEM80 buffer (80 mM Pipes, 4mM MgCl_2_, 1 mM EGTA). Fractions containing DmKHC(1-421)-GFP-6xHis were identified by SDS-page, collected and stored at −80°C in 10% glycerol after snap-freezing in liquid nitrogen.

#### Cell Transfection

COS7 and U2OS cells were plated on 18-mm diameter coverslips 2–4 days before transfection. Cells were transfected with Fugene6 transfection reagent (Roche) according to the manufacturer’s protocol and imaged one day after transfection.

Transfections of hippocampal neurons were performed 24 h before imaging with lipofectamine 2000 (Invitrogen). DNA (1.8 μg per well) was mixed with 3.3 μL lipofectamine 2000 in 200 mL NB, incubated for 30 min, and added to the neurons in NB supplemented with 0.5 mM glutamine at 37°C in 5% CO_2_. After 60-90 min neurons were washed with NB and transferred to the original medium at 37°C in 5% CO_2_ for 1 day.

#### Kinesin Motility Assay

To prepare cellular microtubule cytoskeletons for the kinesin motility assays, the cytoplasm of COS7-cells or hippocampal neurons was extracted for 1 minute in extraction buffer (1M sucrose + 0.15% Triton-X in PEM80) at 37°C. Subsequently, an equal amount of fixation buffer (2% PFA in PEM80 at 37°C) was added and the solution was gently mixed by pipetting for 1 minute. The extraction and fixation buffer were then replaced by washing solution (PEM80 + 100nM Paclitaxel 37°C) for 1 minute. After three more 1-minute washes imaging buffer (1.7% w/v glucose, 185 μg/ml glucose oxidase, 40 μg/ml catalase, 5mM ATP, 1mM DTT, 100mM Paclitaxel in PEM80 buffer at 37°C) was added.

mCherry-tubulin expressing cells were selected for imaging and after a conventional preacquisition of cherry-tubulin, 1 μl of approximately 30nM DmKHC(1-421)-GFP-His was added above the location of acquisition and 10000-20000 frames were acquired at 10 Hz using stream acquisition. Because the concentration of visible kinesins at the selected position gradually decreased because of diffusion and photobleaching, recombinant kinesin was supplemented during imaging to increase the number of localizations of motile kinesins.

For the Nocodazole washout experiments (Figure 1D), COS7 cells were treated with 10 μM nocodazole (M1404, Sigma-Aldrich) for 1 hour at 37°C followed by 1 hour incubation at 4°C. Samples were washed 6x times with cold culturing medium. Subsequently, microtubules were allowed to polymerize for ∼6 minutes at 37°C. Finally, extracted microtubule cytoskeletons were prepared as described earlier. Nocodazole treatments in neurons were performed by using 4 μM nocodazole in the culture medium for 2.5 hours.

Most samples were imaged on a Nikon Ti-E microscope equipped with a 100x Apo TIRF oil immersion objective (NA. 1.49) and Perfect Focus System 3. Excitation was achieved with a mercury lamp or via a custom illumination pathway starting with a Lighthub-6 (Omicron) containing a 638 nm laser (BrixX 500 mW multimode, Omicron), a 488nm laser (Luxx 200 mW, Omicron) and using an optical configuration that allowed tuning the angle of incidence. In most instances, total internal reflection or highly inclined laser illumination was used. Emission light was separated from excitation light with a quad-band polychroic mirror (ZT405/488/561/640rpc, Chroma), a quad-band emission filter (ZET405/488/561/640 m, Chroma), and an additional single-band emission filter (ET525/50 m for GFP emission, Chroma), and detected using a sCMOS camera (Hamamatsu Flash 4.0v2). In some cases, a very similar microscope that has been previously described (Chazeau et al., 2016) was used in the same configuration (see section “Immunocytochemistry, (correlative) SMLM, confocal and gSTED). All components were controlled by Micromanager software (Chazeau et al., 2016).

#### Immunocytochemistry, (Correlative) SMLM, Confocal and gSTED Imaging

Extraction, fixation and immunocytochemistry (ICC) were performed as previously described (Chazeau et al., 2016). Briefly, cells were incubated for 90 s in a extraction buffer preheated at 37°C (80 mM pipes, 2 mM MgCl2, 1 mM EGTA, 0.3% Triton X100 and 0.25% glutaraldehyde, pH 6.9), followed by incubation with 4% PFA preheated at 37°C for 10 minutes. Neurons were further permeabilized with 0.25% Triton X100 and blocking was performed with 2% w/v bovine serum albumin (BSA), 0.2% gelatin, 10 mM glycine, 50 mM NH_4_Cl in PBS, pH 7.4. Primary and secondary antibodies were incubated for 1h at room temperature in blocking buffer. For SMLM (single-molecule localization microscopy), samples were post-fixed in 2% PFA for 10 minutes. Primary and secondary antibodies used in this study are the following: rat monoclonal anti tyrosinated tubulin (Abcam, [YL1/2], ab6160), mouse monoclonal anti acetylated tubulin (Sigma, [6-11B-1], T7451), rabbit polyclonal anti Δ2 tubulin (Millipore, AB3203), mouse monoclonal anti EB1 (BD Bioscience, [clone 5], 610535), Rabbit polyclonal anti GFP (MBL Sanbio, 598),Alexa Fluor 647 Goat Anti-Rat IgG (H+L) (Molecular Probes, Life Technologies A21247), Alexa Fluor 488 Goat Anti-Rat IgG (H+L) (Molecular Probes, Life Technologies A11006), Alexa Fluor 647 Goat Anti-Mouse IgG (H+L) (Molecular Probes, Life Technologies A21236), Alexa Fluor 594 Goat Anti-Mouse IgG (H+L) (Molecular Probes, Life Technologies A11032), Alexa Fluor 568 Goat Anti-Mouse IgG (H+L) (Molecular Probes, Life Technologies A11031), Alexa Fluor 568 Goat Anti-Rabbit IgG (H+L) (Molecular Probes, Life Technologies A11036), Alexa Fluor 594 Goat Anti-Rabbit IgG (H+L) (Molecular Probes, Life Technologies A11032).

Gated STED (gSTED) imaging of acetylated and tyrosinated MTs (Figure 2) was performed with a Leica TCS SP8 STED 3X microscope using a HC PL APO 100x/1.4 oil STED WHITE objective. For excitation of Alexa647 and Alexa594, a pulsed white laser (80MHz) was used at 641 nm and 594 nm, respectively, whereas a 775 nm pulsed laser was used for depletion. Images were acquired in 2D STED mode with the vortex phase mask. Depletion laser power was equal to 35% and 70% of maximum power for Alexa647 and Alexa594, respectively. We used an internal Leica GaAsP HyD hybrid detector with a time gate (tg) of 0.3 ≤ tg ≤ 6 ns and 0.8 ≤ tg ≤ 8 ns for Alexa647 and Alexa594, respectively. Confocal two color imaging was performed on the same setup using the same white laser excitation and emission settings from LAS X controlling software library. Alternatively, for Figures S4C and S4D confocal images were acquired using a LSM 700 confocal laser-scanning microscope (Zeiss) with a 63 × 1.4 N.A. oil objective.

To correlate microtubule orientations and acetylated tubulin using SMLM, motor-PAINT was performed as described above on DIV9/DIV10 neurons. Subsequently the imaged positions were saved and the sample was removed from the microscope but kept in the imaging chamber. To remove the motors from the microtubule lattice the sample was washed two times in PEM80 supplemented with 100 nM paclitaxel and 5 mM ATP. Cells were fixed in 0.3% Glutaraldehyde, 2% PFA and 100 nM paclitaxel in PEM80 for three minutes. After fixation the sample was washed three times in wash buffer (PEM80 with 100 nM paclitaxel) followed by a 30 minute block in blocking buffer (3% BSA, 100 nM paclitaxel in PEM80). Cells were then incubated for one hour with 1/400 mouse anti-acetylated antibody in blocking buffer. After incubation cells were washed three times in washing buffer and incubated for one hour with secondary anti-mouse AlexaFluor647 1/400 in blocking buffer. After three more washes in wash buffer, the buffer was exchanged for imaging buffer (50-100mM MEA, 5% w/v glucose, 700 μg/ml glucose oxidase, 40 μg/ml catalase in PEM80). The sample in the imaging chamber was placed back on the microscope in exactly the same position and the regions where motor-PAINT was performed were imaged by SMLM as described below for Alexa647.

Two color SMLM imaging (Figures S4A and S4B) was performed as previously described (Chazeau et al., 2016) on a Nikon Ti microscope equipped with a 100x Apo TIRF oil objective (NA. 1.49), a Perfect Focus System and an additional 2.5x Optovar to achieve an effective pixel size of 64 nm. Oblique laser illumination was achieved using a custom illumination pathway with a 15 mW 405 nm diode laser (Power Technology), a 50mW 491 nm DPSS laser (Cobolt Calypso) and a 40 mW 640 nm diode laser (Power Technology). Fluorescence was detected using a water-cooled Andor DU-897D EMCDD camera and ET series Cy5 filter (Chroma Technology). All components were controlled by Micromanager software (Edelstein et al., 2010). The composition of the imaging buffer was 100mM MEA, 5% w/v glucose, 700 μg/ml glucose oxidase, 40 μg/ml catalase in PBS buffer. Alexa Fluor 647 and Alexa Fluor A488 were imaged sequentially, using continuous illumination with 640 nm and 491 nm light, respectively. During acquisition, the sample was illuminated with 405 nm light at increasing intensity to keep the number of fluorophores in the fluorescent state constant. Between 10000 and 20000 frames were recorded per acquisition with exposure time of 30/40 ms.

**Table:** 

	Primary Antibodies	Secondary Antibodies	Technique/Microscope
Figures 2D and 2E	Rat anti tyrosinated tubulin Mouse anti acetylated tubulin	Anti Rat Alexa 647 Anti Mouse Alexa 594	Confocal and gSTED/ Leica TCS SP8
Figures 3A and 3B	Rat anti tyrosinated tubulin Mouse anti acetylated tubulin	Anti Rat Alexa 488 Anti Mouse Alexa 568	Confocal/ Zeiss LSM 700
Figure 3F and Figure S5	Mouse anti acetylated tubulin	Anti Mouse Alexa 647	SMLM/ Nikon Ti
Figure S4	Rat anti tyrosinated tubulin Mouse anti acetylated tubulin	Anti Rat Alexa 488 Anti Mouse Alexa 647	SMLM/ Nikon Ti
Figure S4C	Rat anti tyrosinated tubulin Rabbit anti Δ2 tubulin	Anti Rat Alexa 488 Anti Rabbit Alexa 568	Confocal/ Zeiss LSM 700
Figure S4D	Rat anti tyrosinated tubulin Mouse anti EB1	Anti Rat Alexa 488 Anti Mouse Alexa 568	Confocal/ Zeiss LSM 700
Figure 4B and Figure S6B	Mouse anti acetylated Rabbit anti GFP	Anti mouse Alexa 647 Anti rabbit Alexa 594	gSTED/ Leica TCS SP8
Figures 4A and 4B and Figure S6C	Rat anti tyrosinated tubulin Rabbit anti GFP	Anti Rat Alexa 647 Anti rabbit Alexa 594	gSTED/ Leica TCS SP8
Figure S6A	Mouse anti tubulin Rabbit anti GFP	Anti mouse Alexa 647 Anti rabbit alexa 594	gSTED/ Leica TCS SP8
Figures S6D–S6H	Rat anti tyrosinated tubulin Mouse anti acetylated Rabbit anti GFP	Anti Rat Alexa 594 Anti Mouse Alexa 647 Anti rabbit Alexa 488	gSTED/ Leica TCS SP8

#### Simulations

Simulations of motors on microtubule orientations with different ratios of plus and minus end out oriented microtubules were performed as described previously (Kapitein et al., 2010a). In the same reference, we also derived the mathematical expression for the distributions of kinesin-propelled cargoes on different arrays as a function of the fractional orientation probabilities *p*_+_ and *p*_-_ and the average run length before switching microtubules *l*:

$$c\left(x\right)=c_{0}e^{\alpha \;x},\;\text{with}\;\alpha =\frac{p_{+}-p_{-}}{l}.$$

Integrating this to calculate the number of particles *n* at *x*_*n*_ gives

$$n=\frac{1}{\alpha }\left(e^{\alpha \;x_{n}}-1\right).$$

From this, the dendritic coordinate *L*_*50%*_ at which the number of particles before that position equals the number of particles beyond that position can be found by solving

$$\frac{1}{\alpha }\left(e^{\alpha \;L_{50\%}}-1\right)=\frac{1}{2\alpha }\left(e^{\alpha \;L_{dendrite}}-1\right),\;\text{which}\;\text{gives}$$

$$L_{50\%}/L_{dendrite}=\frac{1}{\alpha }\ln\left(\frac{1}{2}e^{\alpha L_{dendrite}}+\frac{1}{2}\right)/L_{dendrite}.$$

#### Single Molecule Localization and Track Orientation Analysis

Images were analyzed using our custom ImageJ plugin called DoM (Detection of Molecules, https://github.com/ekatrukha/DoM_Utrecht), which has been described in detail previously (Chazeau et al., 2016, Yau et al., 2014). Briefly, each image in an acquired stack was convoluted with a two dimensional Mexican hat-type kernel that matches the microscope’s point spread function (PSF) size. Spots were detected by thresholding the images and their sub-pixel positions were determined by fitting a 2D Gaussian function using unweighted nonlinear least-squares fitting with the Levenberg-Marquardt algorithm. Drift correction was applied by calculating the spatial cross-correlation function between intermediate super-resolved reconstructions.

To link localizations into motor trajectories, the linking function of DoM was used as described previously (Sharma et al., 2016). Briefly, linking was performed using a nearest neighbor algorithm were the maximum permitted distance between detected molecules in two subsequent frames, was 3 pixels (∼192 nm). No frame gap was permitted within a track. Only individual tracks that could be observed for at least 3 subsequent frames were included for further analysis. In addition, trajectories in which the angle between two segments was larger than 75 degrees were discarded.

Next, trajectories were separated into different tables based on their direction. For non-neuronal cells, localizations belonging to validated tracks were separated into four different particle tables defined by four criteria for the total displacement in *x* and *y* coordinates of the track (i.e., Δ*x*>0 ∧ Δ*y*>0; Δ*x*>0 ∧ Δ*y*<0; Δ*x*<0 ∧ Δ*y*>0; Δ*x*<0 ∧ Δ*y*<0). For neuronal cells, particle tables were separated into two particle tables corresponding to the axis of the dendrite. The resulting particle tables were subsequently reconstructed using DoM into different super-resolved images that represented all microtubules with similar orientations.

Because the average frame-to-frame displacement of motors was 75 ± 30 nm, tracks appeared as a series of dots when rendered at small pixel sizes. To better visualize these tracks, additional localizations were inserted with a spacing of 15 nm by interpolating between two subsequent localizations within tracks. The localization precision was set as the average of the two observed localizations. Nevertheless, to avoid potential artifacts, all quantifications were performed on the non-interpolated datasets and images.

### Quantification and Statistical Analysis

#### Correlation Analysis

To determine the degree of overlap between images obtained from the retrograde and anterograde runs, the correlation coefficient *C*_*in/out*_ was calculated as

$$C_{in/out}=\frac{\sum_{x=1}^{X}\sum_{y=1}^{Y}i_{in}\left(x,y\right)i_{out}\left(x,y\right)}{\sqrt{\sum_{x=1}^{X}i_{in}^{2}\left(x,y\right)\sum_{y=1}^{Y}i_{out}^{2}\left(x,y\right)}},$$

Where *i*_*in*_(*x*,*y*) and *i*_*out*_(*x*,*y*) are the intensities of the images based on anterograde and retrograde runs at pixel (*x*,*y*), respectively. Similarly, the correlation coefficient *C*_*odd/even*_ was calculated from the intensities *i*_*odd*_(*x*,*y*) and *i*_*even*_(*x*,*y*) of the images based on odd and even localizations within tracks (see Figure S2). The curves of C against different pixel sizes were fitted in Graphpad Prism 5 using the functions described in the legends.

#### Ratio Analysis

Ratios of minus-end out versus minus-end in microtubules were determined based on the intensity of the localizations in either direction. Every detected motor localization in a valid track was normalized to an intensity of 1, so that for both orientations the sum value per image reflected the number of localizations in a specific direction. Subsequently a 5μm segment of a dendrite was selected. These sum values could be presented as arbitrary values along the segment (e.g., Figure 1F) or the total intensity in the minus-end out versus minus-end in was plotted against each other (e.g., Figure 1G) or the ratio of each trace was averaged and used as a single data point (e.g., Figure 1H).

Statistical parameters are included in the figures or corresponding legends. The number of measured segments from different cells is indicated. All quantified data is obtained from at least 3 independent experiments from neurons cultured from different batches. ^∗∗∗^p < 0.001 as tested by a t test or non-parametric Mann-Whitney test.

## Author Contributions

L.C.K. conceived research and supervised the project. R.P.T. optimized motor-PAINT procedures, purified motors, designed and performed experiments, wrote motor-PAINT analysis code, and analyzed data. A.C. optimized procedures for discriminating stable and dynamic microtubules and performed STED and SMLM experiments. B.M.C.C. performed additional experiments and analyzed data. M.L.A.L. created and analyzed the Kif1A-Rigor under the supervision of R.P.T. C.C.H. provided neuronal cultures and gave advice during the project. R.P.T. and L.C.K. wrote the paper with input from all other authors.

## Supplemental Information

Document S1. Figures S1–S7

Movie S1. Video Demonstrating the Real-Time Extraction and Fixation of a COS7 Cell Expressing mCherry-tubulin, Related to Figure 1

Movie S2. Video Demonstrating Motor Movements on the Extracted Microtubule Cytoskeleton of the Zoom of the Cell Indicated in Figure S2, Related to Figures 1 and S2 Imaged at 10 frames/s, total time is 16 s. Scale bar is 1 μm. 4× sped up.

Movie S3. Video Demonstrating Motor Movements on the Extracted Dendritic Microtubule Cytoskeleton of a Hippocampal Neuron, Related to Figure 1E. This video corresponds to Figure 1E. Imaged at 10 frames/s, total time is 16 s. Scale bar is 1 μm. 4× sped up.

Document S2. Article plus Supplemental Information
