# Supplementary material for: Differentiation between Oppositely Oriented Microtubules Controls Polarized Neuronal Transport
Source: Neuron. 2017 Dec 20;96(6):1264–1271.e5. doi: 10.1016/j.neuron.2017.11.018 (PMC5746200; doi:10.1016/j.neuron.2017.11.018)
Supplement: Document S1. Figures S1–S7 [file mmc1.pdf]

Neuron, Volume *96*

Supplemental Information

Differentiation between Oppositely Oriented

Microtubules Controls Polarized Neuronal Transport

Roderick P. Tas, Anaël Chazeau, Bas M.C. Cloin, Maaike L.A. Lambers, Casper C. Hoogenraad, and Lukas C. Kapitein

Figure S1 (related to Figure 1)

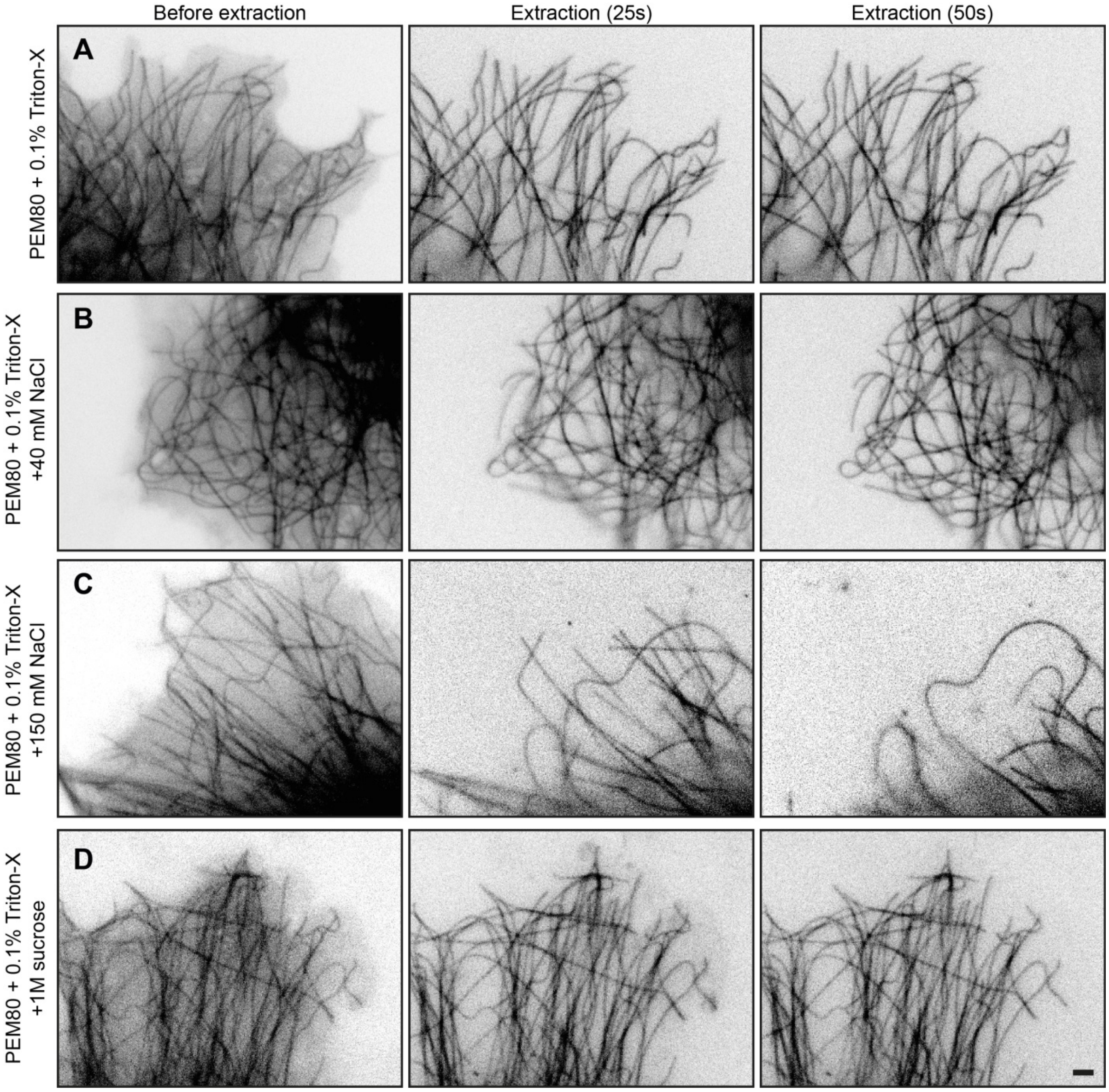

Preservation of the microtubule cytoskeleton during extraction.

Optimization of the extraction protocol to preserve microtubule organization in COS7 cells expressing cherry-tubulin. Images shown were obtained before extraction (left), 25 seconds after extraction (middle), or 50 seconds after extraction and before fixation (right). Extraction was performed in the presence of 0.1-0.15% Triton-X in PEM80 buffer (A), supplemented with NaCl (B,C) or sucrose (D). The conditions in d were used for all subsequent experiments.

Scale bar: 2 μm

Figure S2 (related to Figure 1)

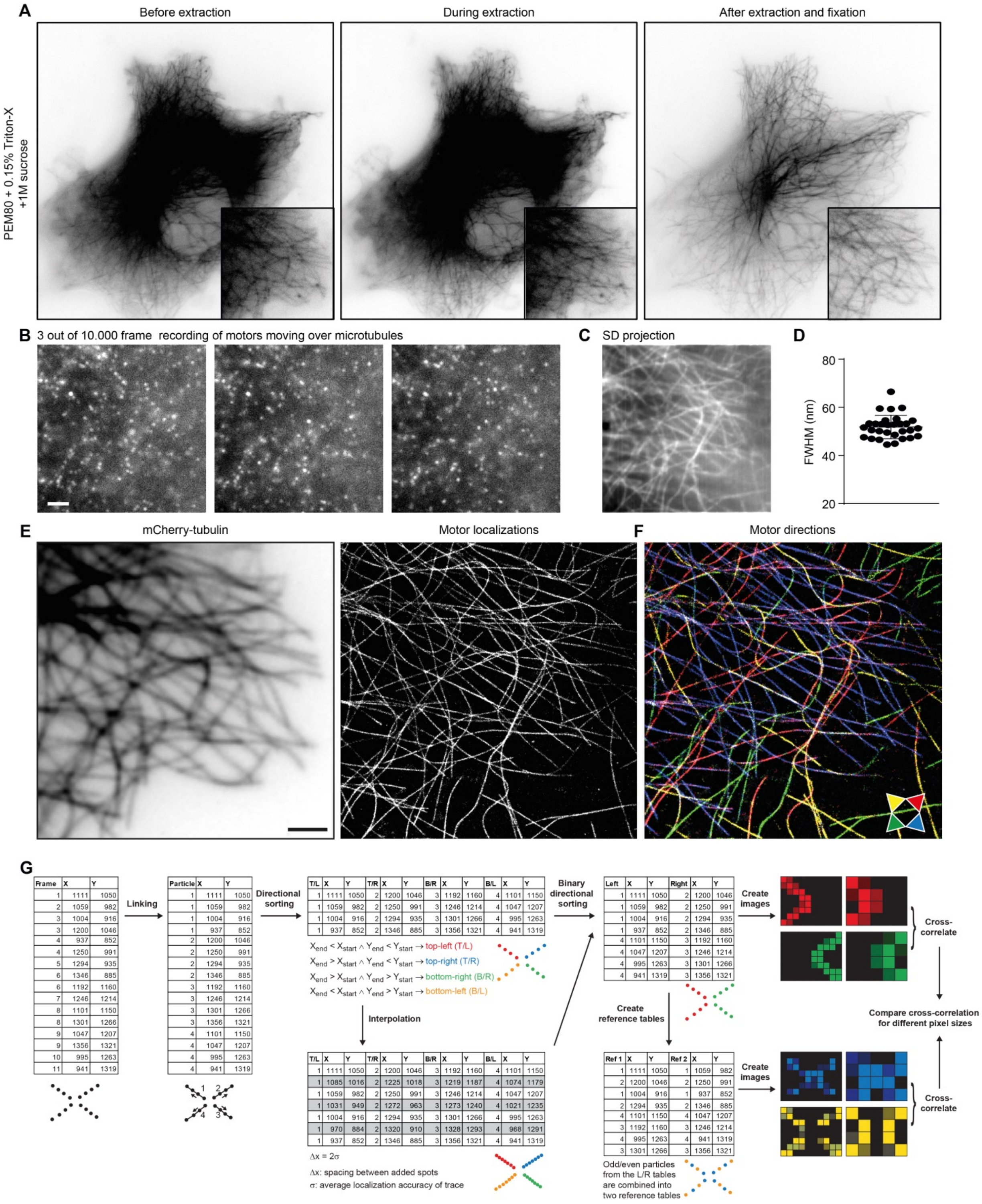

Super-resolution imaging of microtubules and their orientations.

(A) COS7 cell expressing mCherry-tubulin shown before extraction (left), during extraction (middle) and after fixation (right). Zoom indicates the region of interest for which the microtubule orientations are determined in b.

(B) Three subsequent frames from a 10.000 frame recording of the zoom region in a.

(C) Median-filtered (0.3 pixel size) standard deviation projection of the complete 10.000 frame recording of the zoom region in A (also see Supplemental Video 1).

(D) FWHM of cross sections of microtubule imaged by motor tracking (mean±s.d.: 52±1 nm, n=30 profiles).

(E) Zoom of cell shown in A expressing mCherry-tubulin (left) and corresponding super-resolved image obtained by subpixel localization of thousands of motor binding events (middle, 255466 localizations).

(F) Super-resolution reconstruction with all microtubule segments colored according to their absolute orientation. Legend arrows point in the direction of the plus end. Directional image obtained from 19511 motor trajectories with 72846 localizations and created using track interpolation (see methods).

(G) Particle tables containing single molecule information are linked to generate tracks and the resulting tracks are sorted based on the orientation. Optionally, tracks are interpolated to enhance visualization. For neuronal acquisitions tracks are sorted in two bidirectional tables. To analyze the overlap, control particle tables are created by sorting even and odd localizations, irrespective of orientations. Subsequently, the correlation coefficient is calculated for the orientation images or the reference images. (related to Figure 1,2,3,4).

Scale bar: 2 µm

Figure S3 (related to Figure 2)

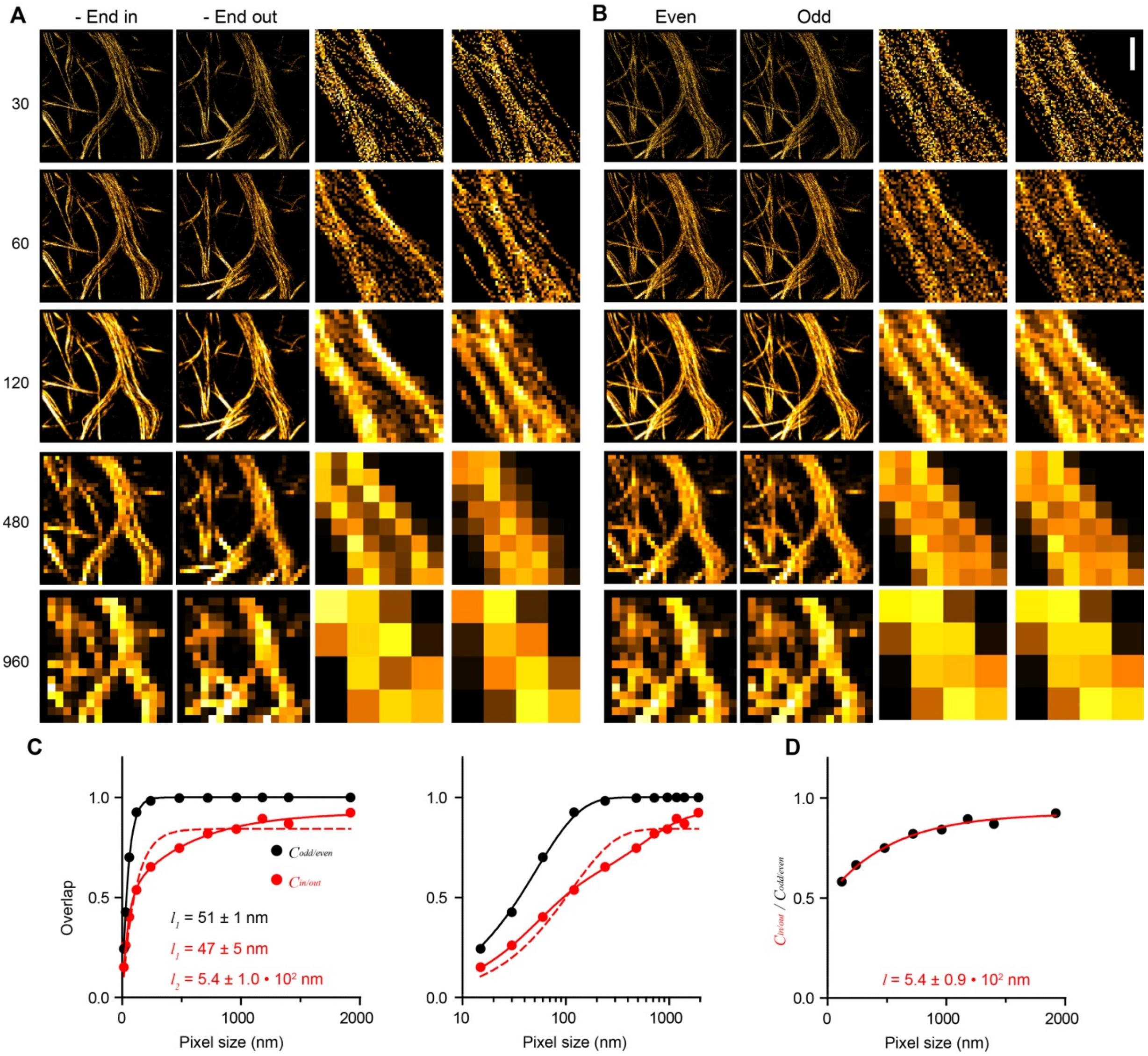

Correlation-based analysis of the spatial extent of orientational order

(A) Motor-based super-resolution reconstructions of a dendritic segment, based on inward runs (left and left zoom) or outward runs (right and right zoom) and rendered at different pixel sizes.

(B) Motor-based super-resolution reconstructions of the same segment, based on all even (left and left zoom) or odd (right and right zoom) localizations, irrespective of direction, and rendered at different pixel sizes.

(C) Degree of overlap as a function of reconstruction pixel size, obtained by calculating the correlation coefficient between reconstructions of minus-end in and minus-end out tracks ($C_{in/out}$), or reconstructions of even and odd localizations, irrespective of direction ($C_{odd/even}$). Solid black and dotted red lines are fits of $C = A(1-\exp(p/l))$, whereas the solid red line is a fit with $C = A(1 - B\exp(p/l_1) - (1-B)\exp(p/l_2))$. Error bars of SE fall within symbol size.

(D) Ratio between $C_{in/out}$ and $C_{odd/even}$ for pixel sizes >100 nm and fitted with $C = A(1-\exp(p/l))$. Error bars of SE fall within symbol size.

Scale bar: 1 μm

Figure S4 (related to Figure 2)

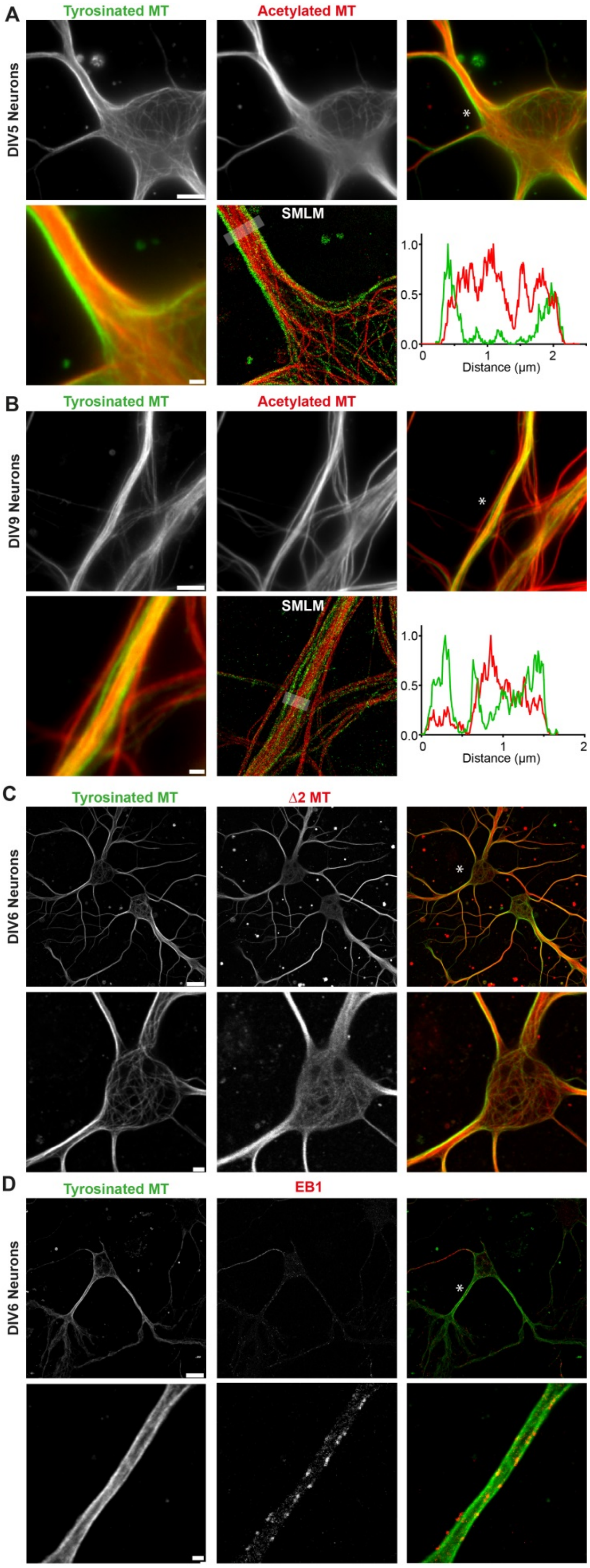

Imaging of tyrosinated, acetylated, Δ2-positive and EB1 decorated MTs in dendrites

(A) DIV5 neurons immunostained for tyrosinated (top left) and acetylated (top middle) tubulin. Top right: merge. A zoom from the merged image (bottom left) is compared to the merged SMLM reconstruction (bottom middle). Bottom right: Intensity profiles from tyrosinated and acetylated MT along the line indicated in the SMLM image. Asterisk marks region for zoom.

(B) Similar to A, but for DIV9 neurons.

(C) DIV6 neurons immunostained for tyrosinated (left) and Δ2 (middle) tubulin. Right: merge.

(D) DIV6 neurons immunostained for tyrosinated tubulin (left) and EB1 (middle). Right: merge.

Scale bar: 5 µm (A, B, top panels), 1 µm (A, B, bottom panels), 10 µm (C, D, top panels), 2 µm (C, D bottom panels).

Figure S5 (related to Figure 3). Minus-end out microtubules are more acetylated

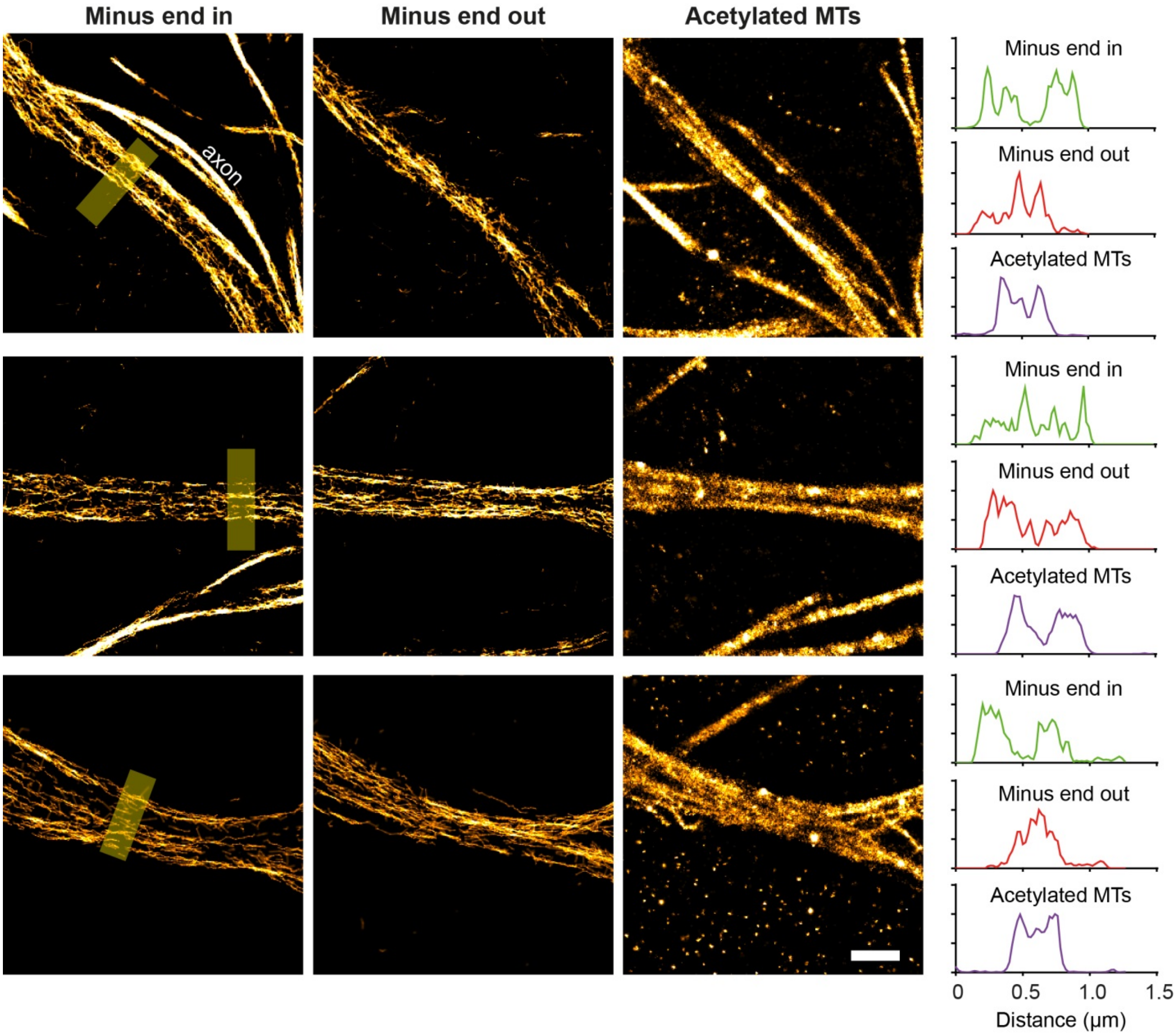

(A) Three additional examples showing correlative motor-PAINT and SMLM for acetylated tubulin. After motor-PAINT cells were stained for acetylated tubulin.

(B) Line scans corresponding to each individual channel in A.

Scale bar: 1 μm

Figure S6 (related to Figure 4). Kinesin-1 and Kinesin-3 prefer different microtubule subsets

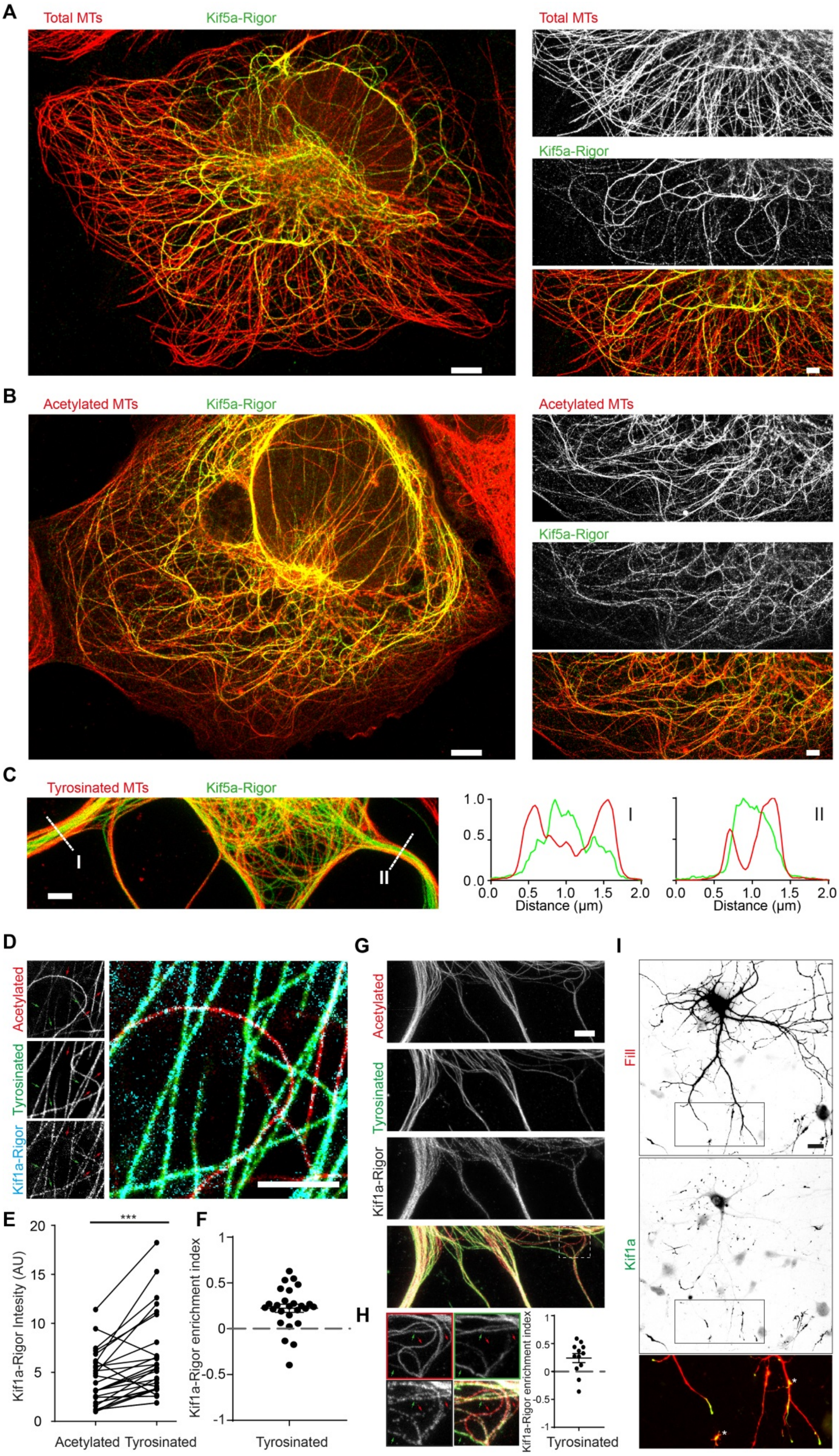

(A,B) STED imaging of COS7 cells after 1 day expression of GFP-kif5a rigor stained for GFP and acetylated-tubulin (A) or GFP and acetylated-tubulin (B). Cellular overview (left) and Zooms of (right) are shown.

(C) Linescans across the minor neurite (I) and longest neurite (II) for the segments shown in Fig. 4A for tyrosinated-tubulin and the kif5a rigor.

(D) STED imaging of COS7 cells after 1 day, low-level, expression of Kif1a-Rigor-GFP stained for GFP, acetylated- and tyrosinated-tubulin. Predominantly acetylated microtubules are indicated by red arrows, tyrosinated microtubules by green arrows.

(E) Paired Kif1a-Rigor intensities on neighboring microtubules that are predominantly tyrosinated compared to acetylated microtubules, within the same imaged region. n= 26 pairs, N= 2, Paired Wilcoxon non-parametric test: ***, $p<0.001$

(F) Kif1a-Rigor enrichment index in COS7 cells for tyrosinated microtubules calculated from the data in (E). Enrichment index was calculated by subtracting the Kif1a-Rigor intensity on acetylated microtubules, I_{ac}, from the intensity on tyrosinated microtubules, I_{tyr}, and dividing by the sum of these signals, i.e. $(I_{tyr} - I_{ac})/(I_{tyr} + I_{ac})$. Positive values mean enrichment on tyrosinated-tubulin, negative values on acetylated microtubules, Mean ± SEM. The mean value of 0.223 corresponds to a 1.58-fold enrichment on tyrosinated microtubules.

(G) STED imaging of DIV 3 neurons after 1 day expression of kif1a Rigor-GFP stained for GFP, acetylated- and tyrosinated-tubulin.

(H) Zoom of region in (G) and corresponding quantification of Kif1a-Rigor microtubule preference. Red arrows indicate exclusively acetylated microtubules, green arrows indicate tyrosinated microtubules. Quantification as in (F) but measured on individual tyrosinated/ acetylated microtubule pairs in neurons. Mean ±SEM, n= 12 pairs, N= 2, paired T-test performed on the raw paired intensities (as in (E)) shows $p = 0.0062$ confirming statistical significance of Kif1a-Rigor preference in neurons. The mean enrichment index of 0.24 corresponds to a 1.63-fold enrichment on tyrosinated microtubules.

(I) DIV 10 neuron overexpressing a mCherry fill and Kif1a-GFP to assess Kif1a localization. Asterisks in zoom indicate axonal tips.

Scale bars: 20 μm (I); 5 μm (A,B overview); 2 μm (A,B,C zooms and D,G)

Figure S7 (related to Figure 4). Orientational order and directional transport

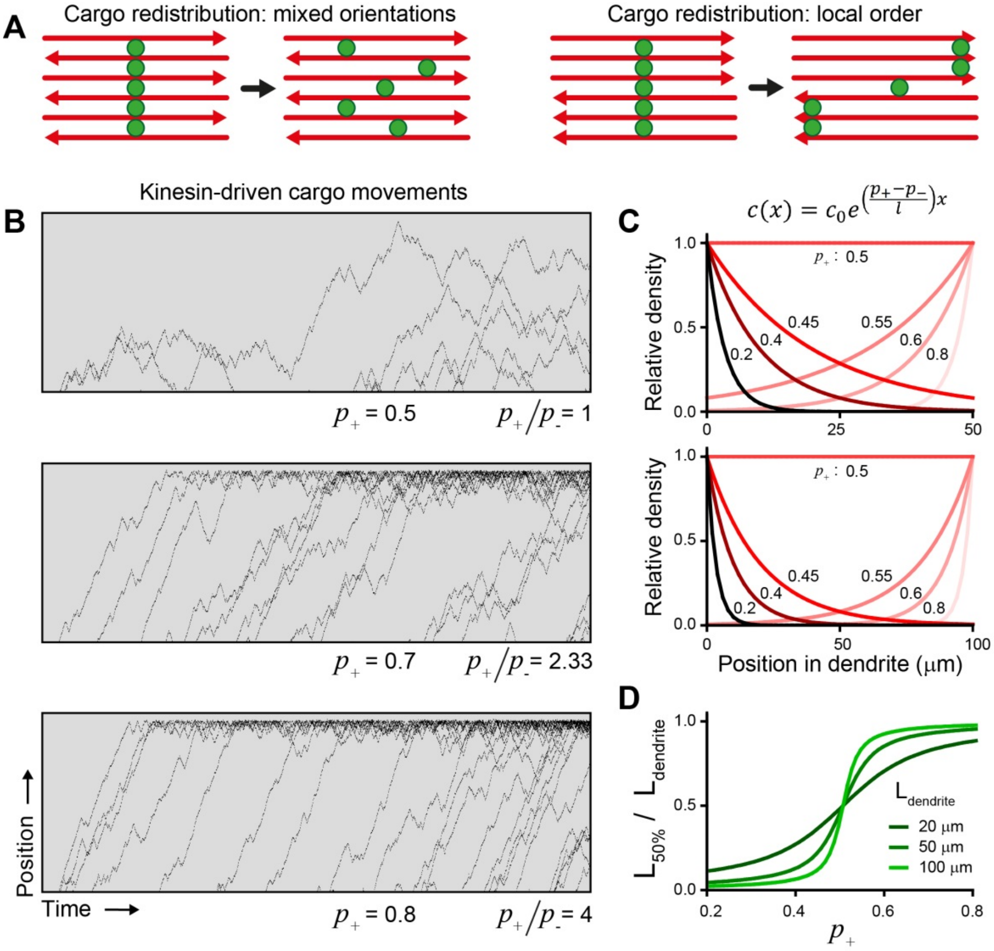

(A) Illustration depicting the influence of microtubule organization on directional transport.

(B) Simulated kymographs of kinesin-driven transport on microtubules arrays with different fractions of plus-end out oriented microtubules p_+, as indicated. A small asymmetry in orientations results in a strong directional bias. Total dendrite length $L_{dendrite}$ is 100 μm and average run length l before selecting a new direction is 2 μm.

(C) Expected distributions of kinesin-driven cargoes for different fractions of plus-end out oriented microtubules p_+ and for three different dendrite length (20, 50, 100 μm). Distributions follow the stated equation and are normalized to maximum density. Numbers indicated in the graph denote the specific p_+ for each curve.

(D) Graph of the relative dendritic coordinate, $L_{50\%}/L_{dendrite}$, at which the number of particles before that position equals the number of particles beyond that position. $L_{50\%}/L_{dendrite}$ is shown against p_+ for three dendritic lengths. Except for p_+=0.5, where $L_{50\%}/L_{dendrite}$ = 0.5, the functional form plotted is $L_{50\%} / L_{dendrite} = \frac{1}{\alpha}\ln(\frac{1}{2}e^{\alpha L_{dendrite}} + \frac{1}{2}) / L_{dendrite}$, with $\alpha = \frac{p_+ - p_-}{l}$.
